# Kaposi‐Juliusberg Varicelliform Eruption complicating Darier disease: May cannabinoid abuse be an underestimated risk factor?

**DOI:** 10.1002/ccr3.4589

**Published:** 2021-08-16

**Authors:** Roberto Russo, Laura Labate, Emanuele Cozzani, Emanuele Delfino, Matteo Bassetti, Aurora Parodi

**Affiliations:** ^1^ Di.S.Sal., University of Genoa Genoa Italy; ^2^ Department of Dermatology IRCCS San Martino Polyclinic Hospital Genoa Italy; ^3^ Department of Infectious Diseases IRCCS San Martino Polyclinic Hospital Genoa Italy

**Keywords:** cannabinoids, Darier disease, HSV, Kaposi‐Juliusberg Varicelliform Eruption

## Abstract

Cannabinoid abuse may facilitate disseminated skin infection by herpes viruses in predisposed patients. These patients should be counselled about that.

## Graphical abstract

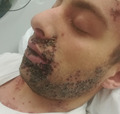

Kaposi‐Juliusberg Varicelliform Eruption (KVE), also called eczema herpeticum, is a disseminated skin infection, attributable to a virus usually causing localized vesicular eruptions, arising in a patient with an underlying cutaneous disease, mainly atopic dermatitis.^1^ More rarely, KVE may occur as a complication of Darier disease (DD).^2^ We report a case of KVE complicating DD, with a possible immunosuppressive role played by cannabinoid abuse.

A 22‐year‐old man was referred to our clinic because of a painful mucocutaneous eruption with odynophagia and dysphagia preventing him from feeding. His personal history was positive for DD, treated with acitretin 25 mg/day, and psychiatric comorbidities including mood disorders and cannabinoid abuse. Physical examination revealed a disseminated vesico‐pustular eruption involving oral and palatal mucosa and the skin of face (mainly lips), trunk, groins and genitalia. (Figures 1 and 2) Some vesicles were umbilicated; others were crusted or eroded. Axillar temperature was 38°C. An esophagogastroduodenoscopy demonstrated esophageal involvement with vesicles. Skin culture was negative for bacteria. Serology for HSV‐1 and 2 was negative. Polymerase chain reaction (PCR) on liquid from vesicular lesions of oral mucosa, lips, and glans tested positive for HSV‐1, confirming the diagnosis of KVE complicating DD. Intravenous acyclovir 5 mg/kg three times a day was administered along with parenteral nutrition. The day after, the patient was afebrile; the eruption and pain gradually improved within days, and the patient was able to feed. The seventh day, anti‐HSV‐1 IgM tested positive.

**FIGURE 1:**
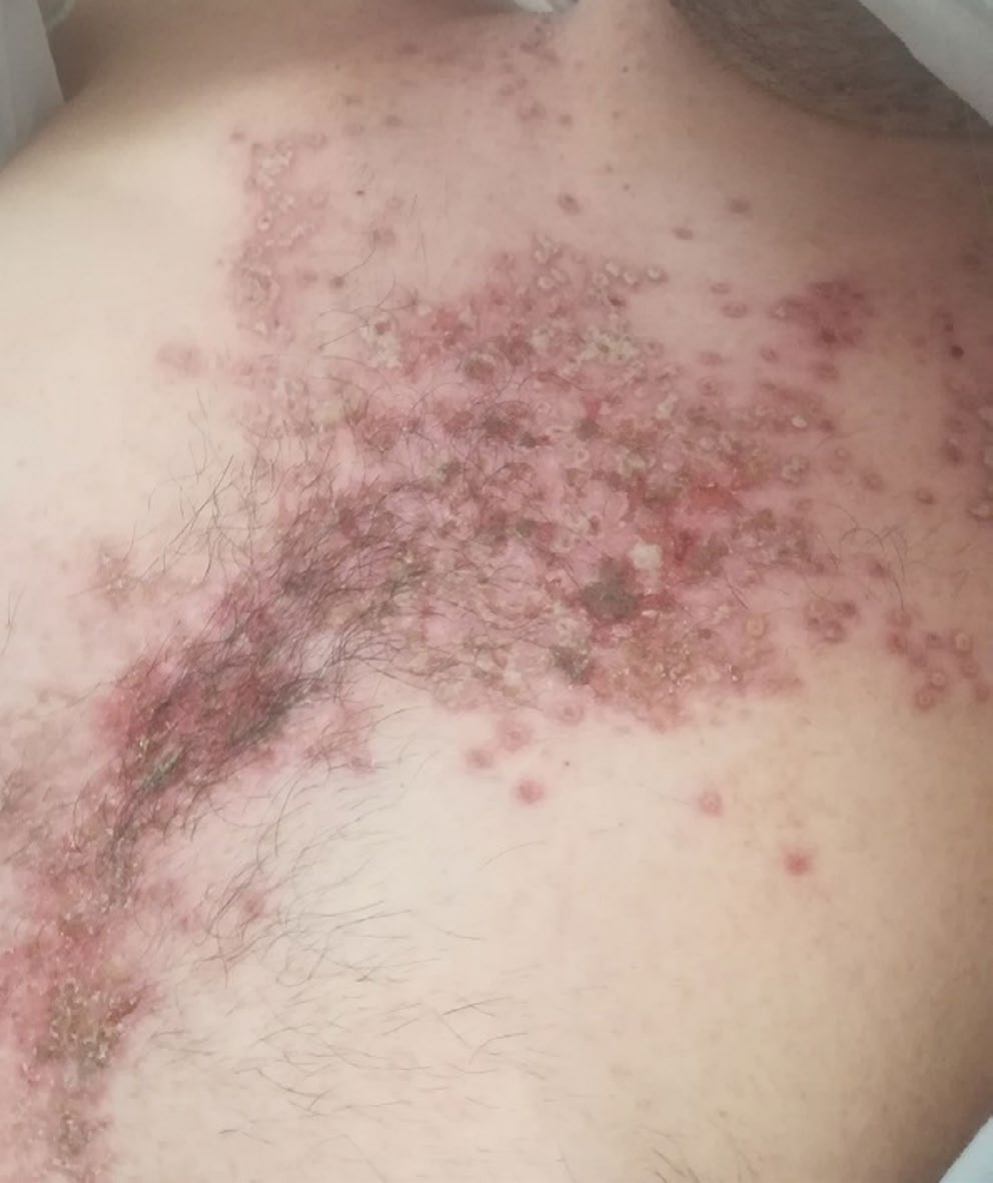
Vesico‐pustular eruption involving chest

**FIGURE 2:**
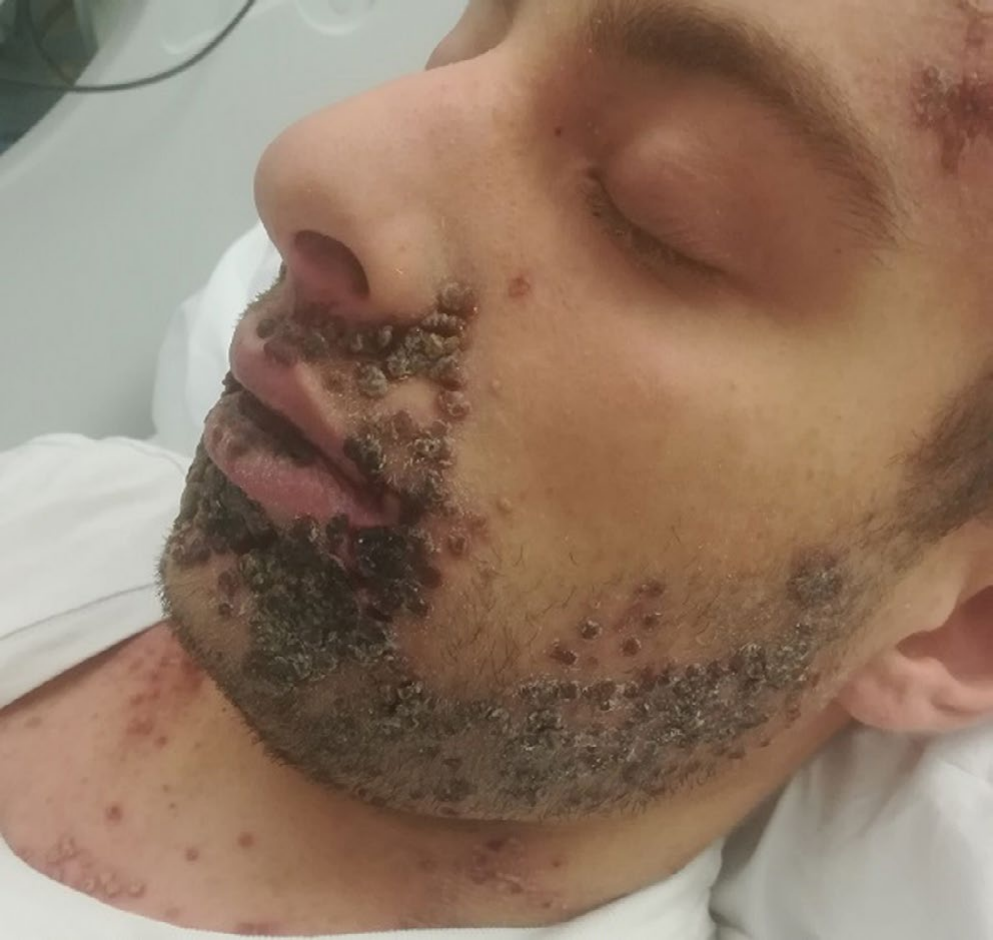
Vesico‐pustular eruption involving face

Though classically associated with atopic dermatitis, KVE complicating acantholytic diseases (such as DD, Hailey‐Hailey disease, and pemphigus) is a well‐known finding. Anyway, it is infrequent to observe a patient with KVE and DD, due to the rarity of DD. A retrospective study on 79 patients with DD reported 11 cases of KVE (14%).^2^

While rare, KVE is a life‐threatening disease. Herpes simplex virus (HSV) is the main etiologic agent (both HSV‐1 and HSV‐2).^1^ In most cases, KVE arises in the genital area and represents a reactivation of the latent herpes virus.^3^ According to the serological tests, in our case, KVE onset seemed to follow first HSV‐1 infection, rather than its reactivation.

A recent in vitro study created an epidermal model with DD‐like barrier dysfunction by silencing ATP2A2 gene: this dysfunction not only resulted in permitting the invasion of HSV‐1 into the deep epidermal layers, but also suppressed the production of interferon‐β and antiviral factors.^4^ Although DD is itself a predisposing factor, we believe that cannabinoids abuse contributed in the development of KVE, by suppressing immune response.

Cannabinoids, through CB1 and CB2 receptors, exert immunosuppressive properties. In fact, they can block leucocytes proliferation, induce apoptosis of T cells and macrophages, and reduce secretion of pro‐inflammatory cytokines.^5^ They also cause malfunction of cytotoxic T lymphocytes (CTLs) by targeting postconjugation of CTL cells.^6^

Furthermore, cannabinoids suppress macrophage extrinsic anti‐HSV function; they impair the splenocyte proliferative response to HSV‐2 and enhance the release of HSV‐2 by disturbing cellular membranes in virus‐infected cells.^7^ Cannabinoids can also mediate the reactivation of latent HSV.^8^

Psychiatric comorbidities, mainly mood disorders, have been clearly associated with DD^9^; therefore, the immunosuppressive influence by cannabinoids may be underrecognized in DD, as the association between mood disorders and substance abuse is well known. Dermatologists should be well conscious of it.

In conclusion, physicians should be very aware of this life‐threatening complication, not only in atopic dermatitis. In fact, KVE is mainly a clinical diagnosis, as systemic antiviral therapy should be started before laboratory confirmation. Additionally, we highlight the need for patients with predisposing skin diseases to avoid immunosuppressive factors, such as ultraviolet radiation exposure or, as in the case of our patient, cannabinoid abuse. We believe that in patients with DD, who often suffer from mood disorders as well, cannabinoid abuse may act as an underestimated immunosuppressive factor.

## CONFLICT OF INTEREST

None declared.

## AUTHOR CONTRIBUTIONS

All authors are contributed in ideation, data collection, draft writing, and final version writing.

## Data Availability

The data that support the findings of this study are openly available (see reference list).
